# Exhaled nitric oxide and clinical phenotypes of childhood asthma

**DOI:** 10.1186/1465-9921-12-65

**Published:** 2011-05-20

**Authors:** Bruno Mahut, Séverine Peyrard, Christophe Delclaux

**Affiliations:** 1 Cabinet La Berma, 4 avenue de la Providence; 92 160 Antony, France; 2 Assistance Publique-Hôpitaux de Paris; Hôpital Européen Georges Pompidou; Service de Physiologie - Clinique de la Dyspnée, Paris, France; 3 Mosquito respiratory research group, Paris, France; 4 University Paris Descartes, Paris, France; 5 CIC 9201 Plurithématique, Hôpital Européen Georges Pompidou, Paris, France

## Abstract

Whether exhaled NO helps to identify a specific phenotype of asthmatic patients remains debated. Our aims were to evaluate whether exhaled NO (FENO_0.05_) is independently associated (1) with underlying pathophysiological characteristics of asthma such as airway tone (bronchodilator response) and airway inflammation (inhaled corticosteroid [ICS]-dependant inflammation), and (2) with clinical phenotypes of asthma.

We performed multivariate (exhaled NO as dependent variable) and k-means cluster analyses in a population of 169 asthmatic children (age ± SD: 10.5 ± 2.6 years) recruited in a monocenter cohort that was characterized in a cross-sectional design using 28 parameters describing potentially different asthma domains: atopy, environment (tobacco), control, exacerbations, treatment (inhaled corticosteroid and long-acting bronchodilator agonist), and lung function (airway architecture and tone).

Two subject-related characteristics (height and atopy) and two disease-related characteristics (bronchodilator response and ICS dose > 200 μg/d) explained 36% of exhaled NO variance. Nine domains were isolated using principal component analysis. Four clusters were further identified: cluster 1 (47%): boys, unexposed to tobacco, with well-controlled asthma; cluster 2 (26%): girls, unexposed to tobacco, with well-controlled asthma; cluster 3 (6%): girls or boys, unexposed to tobacco, with uncontrolled asthma associated with increased airway tone, and cluster 4 (21%): girls or boys, exposed to parental smoking, with small airway to lung size ratio and uncontrolled asthma. FENO_0.05 _was not different in these four clusters.

In conclusion, FENO_0.05 _is independently linked to two pathophysiological characteristics of asthma (ICS-dependant inflammation and bronchomotor tone) but does not help to identify a clinically relevant phenotype of asthmatic children.

## Introduction

Numerous studies have evaluated exhaled nitric oxide (NO) correlates in asthma. For instance, exhaled NO fraction (FE_NO_) has been linked to atopy rather than to asthma *per se *which could be due to the underlying relationship between FE_NO _and eosinophilic inflammation of airways[1]. We and others have emphasized that FE_NO _is also linked to other intrinsic dimensions of asthma such as airway reactivity/tone [2,3] and remodeling of airways[1,4]. All these relationships may explain the complex and still debated relationship between exhaled NO and asthma control/severity[4-6]. Moreover, extrinsic factors also affect FE_NO _such as tobacco exposure and asthma treatment[6,7]. Finally, the epithelial surface of airways, which is linked to the height of the subject and possibly to sex, also affects exhaled NO[8]. Despite this considerable background, the usefulness of its assessment in clinical practice remains debated because of its multidimensional nature, precisely. Furthermore, all these intrinsic and extrinsic exhaled NO modifiers seem to contribute for a minor part of exhaled NO variance,[6] which constitutes a main limitation.

The recent study of Dweik and colleagues has shown that FE_NO _may define an asthma phenotype. They demonstrated that their high FE_NO _phenotype (FE_NO 0.05 _> 35 ppb) was characterized by greatest airway reactivity, airflow limitation, hyperinflation, sputum eosinophilia and levels of symptoms[9]. Nevertheless, FE_NO _levels were similar among patients with severe and non-severe asthma in this latter study. One may hypothesize that the establishment of one or several relationships between FE_NO _and asthma characteristics (either clinical or physiological) is not sufficient to demonstrate that exhaled NO identifies a specific phenotype with clinical relevance, accordingly to a more focused definition of phenotype[10].

The aims of our study were therefore (1) to evaluate the strength of the relationships between exhaled NO and physiopathological asthma characteristics, and (2) to assess whether a specific clinical phenotype can be isolated using exhaled NO measurement. For that purpose, we used two different statistical approaches. The first approach determined the clinical and physiological correlates of exhaled NO, and the degree of FE_NO _variance explained by the correlates. The second approach used a more complex statistical tool, namely cluster analysis, which describes the dimensions of disease without the need for arbitrary *a priori *assumptions about classification, and was specifically designed to test the hypothesis that FE_NO _is associated with a phenotype of childhood asthma.

## Methods

### Design of the study

#### La Berma Cohort

This single centre cohort conducted in a secondary care out-hospital clinic enrolls asthmatic children since 1997. Since 2008, exhaled NO and clinical events were recorded. Levels of asthma control were systematically assessed using only two levels of GINA guidelines during past three months:[11] controlled versus partially/uncontrolled asthma (omitting lung function since PFT were obtained without treatment). Severe exacerbations, according to ATS/ERS definition, [12] and the number of days (1) with symptoms (GINA guidelines) [11] and (2) with systemic steroid were specifically recorded. This cohort has been declared to our regulatory agency for computer data collection (Commission Nationale Informatique et Libertés, n°1408710), and approval from the Ethics Committee of French learned Society of Pulmonology - SPLF was obtained (CEPRO 2009/019). All children and parents were informed of the prospective recording of clinical and physiological data.

#### Patients and criteria of selection from the cohort

We selected a sample of children, meeting the criteria of clinical (episodic symptoms of airflow obstruction with excluded alternative diagnoses) and functional (documented bronchodilator response based on FEV_1 _or sRaw)[13] diagnosis of asthma and who satisfied a full description of their asthma: these 28 variables (see Table 1) are categorized as (1) anthropometrics, (2) past history, (3) parental smoking (more than 5 cigarettes per day), level of control, treatment, and (3) pulmonary function. All data were those specifically determined at the time of only one visit, corresponding to routine evaluation in France. These variables allowed the assessment of three domains of asthma severity: level of current prescribed treatment, level of current baseline control of asthma and immediate past burden of asthma exacerbations, accordingly to Bush and Saglani[14]. The population included in the current retrospective, post hoc, database design study overlaps to some extent with the populations of children published previously[2,6,15-17].

**Table 1:** Clinical and physiological characteristics of the asthmatic children

Characteristics	N = 169	
Sex (male, %)	104 (61%)	
age, years	10.5 ± 2.6	
height, cm	142 ± 15	
weight, kg	38 ± 15	
BMI, kg.m^-2^	18.0 ± 3.5	
**Atopic status (skin prick test)**		
negative	27 (16%)	
1 positive	41 (24%)	
> 1 positive	101 (60%)	
**Tobacco exposure, n (%)**		
maternal	25 (15%)	
paternal	24 (14%)	
paternal and maternal	36 (21%)	
**Clinical events within past 3 months**		
controlled, n (%)	56 (33%)	
partially or uncontrolled	113 (67%)	
number of days with symptoms, median [IQ]	4 [0-12]	
severe exacerbation	42 (31%)	
number of days with systemic steroid, median [IQ]	0 [0-2]	
**Treatment**		
beta-agonist on demand, n (%)	82 (48%)	
low ICS dose, n (%); mean ± SD dose, μg	45 (27%); 154 ± 51	
medium ICS dose, n (%); mean ± SD dose, μg	28 (17%); 357 ± 50	
high ICS dose, n (%); mean ± SD dose, μg	14 (8%); 707 ± 154	
LABA, n (%)	73 (43%)	

**Pulmonary function tests**	**Pre BD**	**Post BD**
sRaw, % predicted	204 ± 45	126 ± 30
FEV_1_, % predicted	97 ± 13	107 ± 12
FEV_1_/FVC, %	78 ± 8	84 ± 6
FVC, % predicted	105 ± 13	108 ± 12
FEF_75-25%_, % predicted	71 ± 19	91 ± 20
FEF_50%_, % predicted	71 ± 18	91 ± 19
TLC, % predicted	104 ± 11	103 ± 10
FRC, % predicted	103 ± 17	100 ± 15
RV, % predicted	107 ± 28	93 ± 22
RV/TLC	0.25 ± 0.06	0.22 ± 0.05
FEF_50%_/TLC	0.71 ± 0.14	0.80 ± 0.18
FENO_0.05 _ppb, median [IQ]	29 [15-48]	

BD: denotes bronchodilator

Results are provided as mean ± SD or median [25^th ^– 75^th ^percentiles: IQ] or absolute number with percent age (%).

### Exhaled NO (FE_NO, 0.05_)

Exhaled NO was measured online, using the Nitric Oxide Analyzer (NIOX; Aerocrine AB; Solna, Sweden: measurement at a constant 50 mL/s expiratory flow rate: FE_NO,0.05_). Measurements were performed according to the ERS/ATS guidelines before pulmonary function tests[18].

### Pulmonary function tests (PFT)

All PFT were performed without inhaled treatment (bronchodilator or LABA/ICS association) on the day of the measurement, by the same operator (BM). Spirometry and plethysmographic measurement of specific airway resistance and thoracic gas volume were performed according to international guidelines and as previously described[13,15,19]. The bronchodilator response to salbutamol 400 μg: (post minus baseline)/baseline FEV_1 _was systematically assessed. Reference values were based on equations edited by Zapletal,[20] as commonly done in Europe[21].

### Statistical analysis

#### First approach

Potential explanatory variable were: age, gender, height, atopy, tobacco exposure, control, treatment and pulmonary function tests. The association between the different explanatory variables and FE_NO _was examined in a multiple linear regression model using the procedure for general linear models with log-transformed FE_NO _values as the dependent variable. The multivariate analysis was performed with a backward selection method and variables with P values of less than 0.05 were retained in the FE_NO _model.

#### Second approach

We used the same approach than Haldar and colleagues[22]. Briefly, a cluster analysis methodology was applied to define homogeneous groups of patients. Principal component analysis (PCA) is a mathematical procedure that uses an orthogonal transformation to convert a set of observations of possibly correlated variables into a set of values of uncorrelated variables called principal components. The number of principal components is usually less than the number of original variables (data reduction). To obtain reliable results, the minimal number of subjects providing usable data for the analysis should be five times the number of variables being analyzed (28 × 5 = 140). This transformation is defined in such a way that the first principal component has as high a variance as possible, and each succeeding component in turn has the highest variance possible under the constraint that it be orthogonal to (uncorrelated with) the preceding components. Then we requested a rotation of the resulting factors which follows completion of the analysis of the data[23]. It has been shown that the relaxed solution of *k*-means clustering, a common method of cluster analysis,[24] specified by the cluster indicators, is given by the PCA principal components, and thus PCA facilitates *k*-means clustering to find near-optimal solutions[25]. Cluster analysis allows the partitioning of data into meaningful subgroups (phenotypes), when the number of subgroups and other information about their composition may be unknown. We hypothesised that FE_NO _measurement could be associated with one of these subgroups of asthmatic children. First, variables for the cluster analysis were selected using a principal component analysis (PCA). When considering variable selection for the cluster analysis, our aims were (1) to choose variables that were measured in clinical practice and contributed to the clinical evaluation of asthma, and to avoid choosing different variables that were representative of the same aspect of the disease as this would introduce further bias when the cluster analysis was performed. We thus performed PCA of our 28 commonly measured clinical variables. Orthogonal varimax rotation was performed and the results are summarized in table 2. To avoid weighting the analysis, we selected only one parameter that was representative of each factor. Two additional variables were also included (see Table 2 legend). Then, a uniform cluster analysis methodology was applied accordingly to Haldar and colleagues (dendrogram for estimation of the number of likely clusters that was further prespecified in a k-means cluster analysis)[22]. Finally, characteristics of clusters were compared using analysis of variance for continuous variables or Kruskal Wallis Rank test (non normal values) and χ2 test for proportions. Statistical analyses were performed using MedCalc 11.3.8 (Mariakerke, Belgium) and OpenStat (version 5) softwares.

**Table 2:** Orthogonal varimax rotation results

28 variables	factors								
	****1****	****2****	****3****	****4****	****5****	****6****	****7****	****8****	****9****

**Clinical**									
gender								**- 0.765**	
age		- 0.879							
height		**- 0.889**							
weight		- 0.905							
BMI		- 0.751							
early wheezing (< 2 years)					- 0.339		0.565		- 0.368
atopy (positive SPT)					0.800				
parental smoking									**0.764**
**Treatment**									
LABA			0.840						
ICS treatment			0.872						
ICS dose			**0.898**						
ICS dose > 200 μg/d			0.814						
**Clinical events**									
partially or uncontrolled				0.439			0.626		
days with symptoms				0.089			**0.777**		
exacerbation				0.942			0.054		
days with oral steroid				**0.940**			0.085		
**Baseline PFT values**									
FEV_1_						0.843			
FEV1/FVC	**0.843**								
sRaw	- 0.609								
RV/TLC		0.645				0.454			
FEF_50%_/TLC	0.834							- 0.050	
**Post bronchodilator PFT values**									
FEV_1_						**0.885**			
FEV1/FVC	0.757								
sRaw	- 0.391								
RV/TLC		0.664							
FEF_50%_/TLC	0.797							0.286	
FEV_1 _response to BD %	- 0.569				0.397			0.392	
**Exhaled NO**	*- 0.061*	*- 0.132*	*- 0.119*	*- 0.059*	**0.789**	*- 0.149*	*0.064*	*0.030*	*0.076*

PFT: denotes pulmonary function tests

All results for exhaled NO are shown in italic for information.

Factor analysis (PCA in our study) is based on the procedure for obtaining a new set of uncorrelated (orthogonal) variables, usually fewer in number than the original set (9 instead of 28 in our study), that reproduces the co-variability observed among a set or original variables. Then we requested a rotation of the resulting factors which follows completion of the analysis of the data. The most common rotation performed is the Varimax rotation[23]. This tends to produce "simple structure", that is, factors which have very high (that are provided in the table) or very low (provided for exhaled NO) loadings for the original variables and thus simplifies the interpretation of the resulting factors.

## Results

Between December 1, 2008 and April 14, 2010, 1001 NO measurements were performed in 592 asthmatic children (clinical diagnose) of which 398 had a positive bronchodilator response in their history. Of these 398 subjects, 169 met the criteria for inclusion in the cluster analysis (all the children fulfilling inclusion criteria were included). Non inclusion criteria were the absence of recent skin pricks test (n = 62), the absence of bronchodilator test on the day of visit (absence of treatment withdrawal, n = 130) and miscellaneous (n = 37). Table 1 shows the characteristics of the 169 asthmatic children.

### First approach

In univariate analysis, FE_NO _was more elevated in atopic than in non atopic children (35[19-57] pbb *versus *12 [9-16] ppb, p < 0.001), less elevated in controlled children (20 [13 - 37] ppb *versus *34 [16-57] ppb, p = 0.018) and more elevated in children with ICS dose ≤ 200 μg/d (19 [13-34] *versus *31 [16-57], p = 0.031). FE_NO _correlated with height (r = 0.29, p < 0.001), age (r = 0.25, p = 0.001), FEV_1 _(r = 0.18, p = 0.021) and bronchodilator response (r = 0.26, p < 0.001). FE_NO _was not decreased by tobacco exposure in univariate analysis. The multivariate analysis demonstrated that 4 variables independently correlated with log FE_NO _(model: r = 0.60, p < 0.001): atopy (r = 0.46, p < 0.001), height (r = 0.29 p < 0.001), bronchodilator response (r = 0.26, p = 0.011) and ICS dose > 200 μg/d (r = - 0.17, p = 0.019). Age, control and forced expiratory flows did not independently contribute to FE_NO _variance.

### Second approach

Orthogonal varimax rotation was performed and the results are summarized in table 2. Based on the pattern of loading we identified the factors as being representative of:

Factor 1: airway obstruction due to increased airway tonus (and airway to lung size ratio)

Factor 2: anthropometrics

Factor 3: treatment

Factor 4: severe exacerbation

Factor 5: atopy

Factor 6: FEV_1 _(airway remodeling)

Factor 7: symptoms

Factor 8: gender

Factor 9: tobacco smoke exposure

Communality of all variables was > 60% (excepting sRaw) and 74.8% of the total variance was explained by the factors (all factors had Eigenvalues > 1). The Kaiser-Meyer-Olkin measure of sampling adequacy was 0.643.

From this result we determined the 9 variables selected for cluster analyses (we favored continuous variables): gender, height, parental smoking, ICS dose, number of days with symptoms and requiring oral steroid, FEV_1_/FVC, FEV_1 _post BD and exhaled NO. Two additional variables were selected (1) FEF_50% _/TLC post-BD (index of airway/lung size) and (2) bronchodilator response (index of airway tonus).

A four-cluster model best fitted the population dataset, which were the following (Table 3): Cluster 1 (47%) described a subgroup of asthmatic boys, unexposed to tobacco, with well-controlled asthma, Cluster 2 (26%) described a subgroup of girls, unexposed to tobacco, with well-controlled asthma (similar to Cluster 1, excepting gender), Cluster 3 (6%) described a subgroup of girls or boys, unexposed to tobacco, with uncontrolled asthma associated with increased airway tone, and Cluster 4 (21%) also described a subgroup of girls or boys, exposed to parental smoking (either father, mother or both), with small airway to lung size ratio and uncontrolled asthma. The only difference (related to gender) between Cluster 1 and 2 was their airway to lung size ratio (p = 0.016, Fisher test).

**Table 3:** Clinical and physiological characteristics according to the four clusters

Characteristics	Cluster 1 n = 79	Cluster 2 n = 44	Cluster 3 n = 11	Cluster 4 n = 35	P value*
Gender, girls/boys ^#^	0/79	44/0	5/6	16/19	< 0.001
Age, years	10.6 ± 2.9	11.0 ± 2.6	10.0 ± 1.3	10.0 ± 2.3	0.34
Height, cm ^#^	144 ± 17	143 ± 14	139 ± 9	139 ± 14	0.33
Weight, kg	39 ± 16	38 ± 16	35 ± 7	35 ± 13	0.46
BMI, kg.m^-2^	18.2 ± 3.2	18.0 ± 4.2	17.8 ± 2.3	17.7 ± 3.4	0.92
Early wheezing, n	33	19	4	12	0.86
Atopy, n (%)	67 (85)	39 (89)	8 (73)	28 (80)	0.55
Tobacco exposure, both ^#^	0	0	1	35	< 0.001
maternal	0	0	0	25	< 0.001
paternal	0	0	1	23	< 0.001
ICS, n (%)	37 (47)	25 (57)	5 (45)	20 (57)	0.62
ICS dose, BED μg/d ^#^	135 ± 208	180 ± 236	149 ± 199	188 ± 217	0.57
LABA, n	33 (42)	22 (50)	4 (36)	15 (43)	0.58
**Clinical events**					
Partially or uncontrolled, n	46 (58)	29 (66)	10 (91)	28 (80)	0.028
Days with symptoms ^#^	3 [0-8]	4 [0-12]	3 [3-7]	7 [3-15]	0.045
With exacerbation, n	16 (20)	6 (14)	6 (55)	14 (41)	0.006
Days with oral steroid ^#^	0 [0-0]	0 [0-0]	3 [0-3]	0 [0-3]	0.021
**Pulmonary function tests**					
*Before bronchodilation*					
**FE_NO_, ppb **^#^	25 [14-45]	34 [19-51]	21 [9-49]	30 [14-52]	**0.58**
sRaw, % pred	201 ± 40	200 ± 54	211 ± 37	212 ± 47	0.68
FEF_50%_/TLC	0.70 ± 0.13	0.74 ± 0.13	0.62 ± 0.15	0.69 ± 0.14	0.5
FEV_1_, % pred	98 ± 13	100 ± 13	91 ± 14	97 ± 14	0.28
FVC, % pred	104 ± 14	106 ± 13	106 ± 15	106 ± 12	0.88
FEV_1_/FVC ^#^	78 ± 8	80 ± 6	74 ± 8	77 ± 9	0.41
FEF_25-75%_, % pred	74 ± 18	72 ± 18	59 ± 19	70 ± 20	0.1
TLC, % pred	105 ± 12	104 ± 9	103 ± 12	106 ± 10	0.32
FRC, % pred	103 ± 17	102 ± 17	101 ± 22	105 ± 18	0.22
RV, % pred	107 ± 28	104 ± 27	101 ± 20	115 ± 32	0.23
RV/TLC	0.24 ± 0.06	0.25 ± 0.06	0.24 ± 0.04	0.27 ± 0.06	0.14
*After bronchodilation*					
sRaw, % pred	125 ± 28	127 ± 31	117 ± 22	133 ± 34	0.22
FEF_50%_/TLC ^#^	0.78 ± 0.17	0.86 ± 0.15	0.80 ± 0.22	0.75 ± 0.22	0.025
FEV_1_, % pred ^#^	107 ± 12	110 ± 12	105 ± 14	107 ± 12	0.47
FVC, % pred	107 ± 13	108 ± 12	107 ± 14	110 ± 12	0.69
FEV_1_/FVC, %	84 ± 6	86 ± 4	83 ± 8	82 ± 7	0.05
FEF_25-75%_, % pred	92 ± 20	95 ± 16	86 ± 25	84 ± 21	0.17
TLC, % pred	103 ± 11	102 ± 9	101 ± 13	104 ± 10	0.73
FRC, % pred	101 ± 16	100 ± 14	95 ± 19	101 ± 13	0.3
RV, % pred	95 ± 22	90 ± 22	91 ± 25	96 ± 23	0.78
RV/TLC	0.22 ± 0.05	0.22 ± 0.05	0.23 ± 0.04	0.23 ± 0.05	0.6
Bronchodilator response, % ^#^	8 [5-12]	10 [5-17]	15 [9-20]	8 [3-16]	0.036

# : variables included in the cluster analysis

* : Comparison between clusters using analysis of variance for continuous variables or Kruskal Wallis Rank test (non normal values) and χ^2 ^test for proportions. Significance values for variables included in the cluster analysis are a product of the cluster algorithm and are provided for illustrative purposes only.

Results are provided as mean ± SD or median [25^th ^- 75^th ^percentiles] or absolute number with percent age (%).

## Discussion

The main results of our study is the demonstration that single-flow rate exhaled NO (FENO_0.05_) is independently associated with two main asthma pathophysiological characteristics, namely airway inflammation and airway tone, but that FENO_0.05 _does not help to distinguish a relevant clinical phenotype of childhood asthma in a cross-sectional assessment.

### Design issues

We specifically assessed different components of asthma control definition, such as symptoms and exacerbations because whether severe exacerbation constitutes the ultimate expression of loss of control and/or a more unpredictable even remains controversial[12,14]. Table 2 further showed that control and exacerbations were two different dimensions of asthma, which could be related to our pediatric population[14]. Only two levels of control were assessed (controlled versus partially/uncontrolled patients) because the achievement of control is the main clinical issue. Our percentage of controlled children (33%) is in accordance with a French cross-sectional study in childhood asthma[26]. Since a dose effect of ICS on exhaled NO can be demonstrated,[2] we used it as an indirect index of airway inflammation, which is an obvious short-cut inasmuch as the explained variance of FE_NO _by ICS is only ~3% in our study. Sputum eosinophilia can be regarded as the gold standard measure of inflammation but is not routinely assessed in most centres. The recent study of Schleich and colleagues demonstrates that FE_NO _is able to identify a sputum eosinophil count ≥ 3% with reasonable accuracy and thresholds which vary according to dose of ICS[27]. Finally, we deliberately chose to add two variables in the cluster analyses, namely post-bronchodilator FEF_50% _/TLC and bronchodilator response. The former is an index of airway size/lung size[28]. We hypothesized that such an index, which has been linked to the risk of airway responsiveness,[29] may influence symptoms. The latter may also be an important pathophysiological characteristic of asthma associated with poor clinical outcomes in childhood asthma[30].

Four clusters were identified in our pediatric population. The first two clusters can be considered similar when excluding gender and corresponds to the most prevalent group of asthmatic children in an out-hospital specialized clinic (73%, 123/169) with 75/123 children having partially/uncontrolled asthma. The remaining two clusters (27%, 46/169) were constituted of boys and girls with a more severe (or undertreated) disease (38/46 with partially/uncontrolled asthma, 20/46 with a recent exacerbation). Interestingly, these two clusters only differ by their underlying severity factors, namely increased airway tonus (cluster 3, 6% of the population) and parental tobacco exposure while having small airway/lung size ratio (cluster 4, 21%). The prevalence of cluster 3 is similar to that of difficult-to-treat patients (~5%), and may also constitute an asthma phenotype characterized by increased airway tone and lability[14,30]. The fourth cluster segregates children exposed to passive smoking that have a more severe disease, which is in line with the results of a French cross-sectional study in 3431 children demonstrating that unacceptable asthma control was associated with passive exposure to parental tobacco smoke[26]. Overall, the phenotypes that have been identified by the cluster analysis can be a posteriori explained, which further validate the statistical approach to assess exhaled NO usefulness. It has to be emphasized that the degree of asthma control did not clearly differentiate the clusters in our study. Several explanations can be discussed. Firstly, our study deals with childhood asthma, this specific population is often partially controlled because some degree of exercise limitation (or symptoms) is often present and exacerbations are often unpredictable events, mostly related to viral infections [31,32]. Secondly, normal lung function (under treatment) is the rule in asthmatic children [15,17] and a « phenotype » of children exhibiting a decline in lung function is almost impossible to isolate[17]. Thirdly, therapeutic compliance (and inability to use the inhaler properly) in children may be difficult to obtain [33] that may further explain the presence of mild symptoms.

### Usefulness of FENO_0.05 _measurement

We confirm by our first statistical approach that FE_NO _is linked to its classical modifiers such as height and atopy. The link with ICS dose is more controversial, but we previously evidenced such a link with a plateau effect of ICS[6]. We show that exhaled NO and bronchodilator response are linked, a result that was previously obtained by different research groups[2,3,9]. More interestingly, we demonstrate for the first time that the relationships between exhaled NO and both ICS dose (an indirect marker of airway inflammation) and bronchodilator response (a marker of airway tonus) are independent. Exhaled NO measurement is claimed to be an allergic inflammometer, but it is also a marker of airway smooth muscle tone[34].

The recent study of Dweik and colleagues has shown that a high FE_NO _"phenotype" (FE_NO 0.05 _> 35 ppb) was characterized by greatest airway reactivity, airflow limitation, hyperinflation, sputum eosinophilia and levels of symptoms[9]. Consequently, our results (first statistical approach) are in agreement with their data, but we further suggest that FE_NO _is not specifically associated with a clinically relevant phenotype in asthmatic children. Haldar and colleagues elegantly demonstrated that eosinophilic inflammation helps to characterize adult asthma phenotypes[22]. Consequently our results may seem at variance, but exhaled NO and airway eosinophilia could be discordant in childhood[14]. In summary, to our best knowledge, this is the first study in childhood asthma, showing that exhaled NO does help to describe a clinically useful phenotype despite its ability to describe underlying pathophysiology (inflammation and modified airway smooth muscle function).

### Limitations of the study

Principal among these is the cluster analysis methodology. The use of an algorithm that separates the population into discrete clusters may not be realistic. The limited size of our out-hospital population, the restricted analysis of childhood asthma (that could be a more homogeneous disease per se), our choice of clustering parameters and the loss of clinical material through attrition of the data set may have introduced some bias, but we hypothesized that the clinical relevance of exhaled NO should be "easily" demonstrated. It has to be stated that, in a cross-sectional design mainly assessing asthma control, FENO0.05 was not associated with a specific cluster, which is in accordance with recent trials failing to demonstrate the clinical usefulness of this measure for asthma control[35].

### Perspectives

It has to be emphasized that, in a cross-sectional design mainly assessing asthma control, FENO_0.05 _was not associated with a specific cluster, which is in accordance with recent trials failing to demonstrate the clinical usefulness of this measure for asthma control[35]. Whether peripheral airway/alveolar NO concentration after correction for axial NO back-diffusion, which is elevated in a subset of asthmatic patients (~25%), could help to identify a specific "phenotype" of asthma warrants further studies[4,36,37].

In conclusion, FENO_0.05 _is independently linked to two pathophysiological characteristics of childhood asthma (ICS-dependant inflammation and bronchomotor tone) but does not help to identify a clinically relevant phenotype of asthmatic children in a cross-sectional analysis of routinely recorded parameters.

## Competing interests

The authors declare that they have no competing interests.

## Authors' contributions

BM carried out the measurements, participated in the design of the study and drafted the manuscript. SP performed the statistical analysis and helped to draft the manuscript. CD2 conceived of the study, participated in its design and helped to draft the manuscript. All authors read and approved the final manuscript.
